# Prevalence of neonatal near miss and associated factors in Nepal: a cross-sectional study

**DOI:** 10.1186/s12884-021-03894-3

**Published:** 2021-06-09

**Authors:** Rajbanshi Sushma, Mohd Noor Norhayati, Nik Hussain Nik Hazlina

**Affiliations:** 1 Women’s Health Development Unit, School of Medical Sciences, Universiti Sains Malaysia, Health Campus, 16150 Kubang Kerian, Kelantan, Malaysia; 2 Department of Family Medicine, School of Medical Sciences, Universiti Sains Malaysia, Health Campus, 16150 Kubang Kerian, Kelantan, Malaysia

**Keywords:** Neonatal near miss, Neonatal morbidity, Severe maternal morbidity, Cross-sectional study, Nepal

## Abstract

**Background:**

The rate of neonatal mortality has declined but lesser than the infant mortality rate and remains a major public health challenge in low- and middle-income countries. There is an urgent need to focus on newborn care, especially during the first 24 h after birth and the early neonatal period. Neonatal near miss (NNM) is an emerging concept similar to that of maternal near miss. NNM events occur three to eight times more often than neonatal deaths. The objective of this study was to establish the prevalence of NNM and identify its associated factors.

**Methods:**

A hospital-based cross-sectional study was conducted in Koshi Hospital, Morang district, Nepal. Neonates and their mothers of unspecified maternal age and gestational age were enrolled. Key inclusion criteria were pragmatic and management markers of NNM and admission of newborn infants to the neonatal intensive care unit (NICU) in Koshi Hospital. Non-Nepali citizens were excluded. Consecutive sampling was used until the required sample size of 1,000 newborn infants was reached. Simple and multiple logistic regression was performed using SPSS® version 24.0.

**Results:**

One thousand respondents were recruited. The prevalence of NNM was 79 per 1,000 live births. Severe maternal morbidity (adjusted odds ratio (aOR) 4.52; 95% confidence interval (CI) 2.07–9.84) and no formal education (aOR 2.16; 95% CI 1.12–4.14) had a positive association with NNM, while multiparity (aOR 0.52; 95% CI 0.32–0.86) and caesarean section (aOR 0.44; 95% CI 0.19–0.99) had negative associations with NNM.

**Conclusions:**

Maternal characteristics and complications were associated with NNM. Healthcare providers should be aware of the impact of obstetric factors on newborn health and provide earlier interventions to pregnant women, thus increasing survival chances of newborns.

## Background

The rate of under-five mortality has long been considered an important indicator of social development, economic prosperity and healthcare quality. Globally, a 51% decline in neonatal mortality was recorded between 1990 and 2017; however, the decline in neonatal mortality has been slower than that of post-neonatal under-five mortality [1]. Annual neonatal mortality rates range from 0.9 to 44.2 deaths per 1,000 live births [1]. South Asia had 25 neonatal deaths per 1,000 live births in 2018 and is a hub of the highest number of neonatal deaths along with sub-Saharan Africa [1, 2]. A child born in Asia is 10 times more likely to die in the first month of life than a child born in high-income countries [2]. The objective of Sustainable Development Goal 3 (SDG3) and the global Every Newborn Action Plan is to reduce neonatal mortality to ≤ 10 per 1,000 live births by 2030 [3].

The neonatal mortality rate in Nepal was 21 per 1,000 live births in 2016; about four-fifths (79%) of all neonatal deaths were early neonatal deaths and 57% of all births were institutional births [4]. There are large variations in neonatal mortality within provinces of Nepal from 15 to 41 per 1,000 live births. Nepal needs to reduce its neonatal mortality rate by half in the next 10 years to achieve SDG3.2. Accelerated efforts are thus needed to address interprovincial disparities. Neonatal near miss (NNM) is a novel concept that has recently emerged and is similar to maternal near miss (MNM). It provides vital information required for evaluation of the quality of care in hospitals and explores opportunities to improve the performance of healthcare providers [5]. Near-miss events occur three to eight times more often than neonatal deaths [6, 7]. Thus, NNM evaluations can provide abundant evidence of the causal pathways responsible for neonatal deaths [8].

Conceptualization of the term “NNM” in 2009, similar to “MNM”, was proposed by Avenant [9]. That same year, Pileggi et al*.* established pragmatic NNM criteria using 2005 WHO Global Survey data [10]*.* The initial definition of pragmatic markers included very low birth weight (< 1,500 g), gestational age at birth (< 30 weeks) or Apgar score (< 7 at five minutes) and surviving seven days after birth [10].

Pileggi-Castro et al*.* re-evaluated the NNM definition using WHO Global Survey data and validated the revised definition using the WHO Multi-Country Survey on Maternal and Newborn Health data [10–12]. NNM refers to “neonates that nearly died but survived severe complications at birth or during the neonatal period” [10, 12]. The recommended pragmatic criteria were birth weight below 1,750 g, gestational age < 33 weeks or Apgar score < 7 at five minutes in newborn infants who survive for seven days after birth. Whereas for diagnostic accuracy, management markers from this definition included the use of therapeutic intravenous antibiotics, nasal continuous positive airway pressure, intubation, phototherapy within the first 24 h, cardiopulmonary resuscitation, vasoactive drugs, anticonvulsants, surfactant administration, blood products, steroids to treat refractory hypoglycemia and surgery in early neonatal life [13]. The pragmatic criteria and management markers developed by Pileggi-Castro et al*.* were shown to have a sensitivity of 93% and specificity of 97% [13].

There is no uniform definition of NNM to this date, although a vast number of NNM studies are available. Systematic reviews on NNM, conducted in 2015 and 2017, recommended developing a standard definition for NNM [14, 15]. Worldwide prevalence of NNM ranged from 39.2 to 131 per 1,000 live births in 2014 and 2018 [16, 17]. A population-based study conducted in Nepal applied community-appropriate NNM criteria adapted from Pileggi et al. [10] and adjusted to the local context, demonstrated a prevalence of 22 per 1,000 live births [18]. NNM was shown to be caused by birth asphyxia (70%), very low birth weight (17%), neonatal sepsis (10%) and prematurity (3%) [18].

The NNM definition proposed by Pileggi-Castro et al*.* is used in this study. Researchers assessing NNM in South Asia have applied pragmatic criteria only [19, 20]. Thus, the current study is the first in Nepal to use a combination of pragmatic and management criteria to establish NNM prevalence and identify its associated factors. In this study, NNM referred to “an infant who nearly died but survived a severe complication that occurred during pregnancy, birth or within seven days of extrauterine life” [13]. Shifting the focus from neonatal mortality towards NNM and associated factors can be useful information for policymakers to improve neonatal care.

## Methods

This cross-sectional study was conducted on 1000 newborns and their mothers admitted to the postnatal ward in Koshi Hospital, Morang district, Nepal. Morang district was chosen based on its high population density and diverse ethnicity. Koshi Hospital is a referral hospital for the eastern part of Nepal and has a neonatal intensive care unit (NICU). It is located in Biratnagar city of Morang district and is the second-most densely populated city in Nepal with a population of 1,073,307 and 27,799 expected pregnancies annually [21]. The hospital has 35 beds in the postnatal ward and manages approximately 9,000 annual births. The NICU contains six beds and admits approximately 45 neonates per month.

Mothers of any age and newborns at any gestational age who survived seven days after birth were included. Non-Nepali citizens and non-Morang residents were excluded. Consecutive sampling was applied. The sample size was calculated based on the prevalence of NNM using a single proportion formula. With NNM prevalence of 2.2%, a precision of 0.01 and a 20% non-response rate, the calculated sample size was determined to be 1,000 newborns [18].

The research tool comprised of maternal and neonatal hospital records and socio-demographic information. Two recent nursing undergraduates collected the data daily. The hospital’s records on obstetric history, pregnancy complications and data of the newborns were extracted into a case report form on the day of discharge of the mother. Newborns in NICU were followed up daily and their information was updated after discharge. Socio-demographic information was obtained from the mothers using face-to-face interviews.

Data were entered and analyzed using IBM SPSS® Statistics 24.0. Frequencies and percentages were calculated for categorical variables; mean, median, standard deviation and interquartile range were determined for the numerical variables. Simple and multiple logistic regression was used to assess associated factors. Clinically significant variables in simple logistic regression analysis and those with *p*-value < 0.3 were included in the multiple logistic regression analysis. Adjusted odds ratio (aOR), corresponding 95% confidence interval (CI), and *p*-value < 0.05 were calculated and considered statistically significant.

The outcome variable was NNM status [13]. The independent variables were ethnicity, religion, wealth index, place of residence, mother’s and father’s education, mother’s and father’s occupation, father’s smoking habit, mother’s age, age of marriage, duration of marriage, parity, number of antenatal care visits (ANC), self-reported pre-pregnancy body mass index, mode of birth, obstetric hemorrhage, hypertensive disorders of pregnancy, other maternal systemic disorders, severe management indicators (clinical management such as blood transfusion, central venous access, hysterectomy, intensive care unit admission, prolonged hospital stay, intubation not related to the anesthetic procedure, return to operating room and laparotomy), severe maternal morbidity (SMM) and sex of the newborns.

## Results

One thousand newborns and their mothers were recruited in the study between November 2019 and March 2020. There were 18 perinatal deaths (17 stillbirths and one early neonatal death) during the study period. There were 10 multiple births, and these were treated as a single birth based on the first twin birth. Of these, four were NNM cases.

The prevalence of NNM was 79 per 1,000 live births in Koshi Hospital (Table 1). Table 1 shows pragmatic (*n* = 65) and management markers (*n* = 27) used to evaluate NNM. The NNM markers were found overlapping in newborns. The most frequently encountered pragmatic criterium was Apgar score < 7 at five minutes after birth (41/65; 63.1%) followed by birth weight < 1,750 g (20/65; 30.7%). All three pragmatic criteria applied to only one newborn. Of the 65 neonates with pragmatic markers, 35 (53.9%) required NICU admission and of the 27 admitted in NICU with management markers, 24 (88.9%) had at least one pragmatic criterium.

**Table 1:** Pragmatic and management criteria of NNM in Koshi Hospital

NNM characteristics	n	(%)
**Pragmatic criteria**		
APGAR Score^a^ < 7 in 5 min	41	(63.1)
Birth weight < 1750 g	20	(30.7)
Gestational age < 33 weeks	11	(16.9)
Any pragmatic marker of severity	65	(100.0)
**Management criteria**		
Use of therapeutic antibiotics	25	(92.6)
Nasal continuous positive airway pressure	19	(70.3)
Cardiopulmonary resuscitation	11	(40.7)
Use of phototherapy in the first 24 h	6	(22.2)
Any intubation (anytime within the first week)	3	(11.1)
Use of anticonvulsants	2	(7.4)
Use of steroids to treat refractory hypoglycemia	1	(3.7)
Surfactant administration	0	(0)
Use of a vasoactive drug	0	(0)
Use of any blood products	0	(0)
Any surgery	0	(0)
Admission to NICU with any management marker of severity	27	(100.0)
Overall NNM^b^	79	(7.9)

^a^APGAR Score: Appearance, Pulse, Grimace, Activity, and Respiration

^b^79 newborns with NNM had 92 NNM criteria

Newborns admitted to NICU were 44, but 17 were referred to NICU in private hospitals after birth in Koshi Hospital. Therefore, hospital records could be accessed for only 27 newborns in Koshi Hospital (Table 1). In the NICU, 25 (92.6%) were treated with therapeutic antibiotics and 19 (70.3%) with nasal continuous positive airway pressure.

Socio-demographic and maternal characteristics with and without NNM are depicted in Table 2. In addition, the proportion of adolescent mothers (< 20 years) was higher for NNM (17/79; 21.5%) as compared to those without (93/921; 10.1%).

**Table 2:** Socio-demographic and maternal characteristics based on NNM status

Variables	Neonatal Near Miss (*n* = 79)				non-Neonatal Near Miss (*n* = 921)			
	Mean	SD	n	(%)	Mean	SD	n	(%)
**Socio-demographic**								
Mother’s age (year)^a^	21	(20, 24)			22	(20, 25)		
Age of marriage (year)	18.91	2.43			19.27	2.59		
Duration of marriage (year)^a^	2	(1, 4)			2	(1, 6)		
Ethnicity								
Muslim			16	(20.3)			86	(9.3)
Terai/Madhesi			28	(35.4)			381	(41.4)
Dalits			16	(20.3)			163	(17.7)
Janajati			15	(19.0)			200	(21.7)
Brahmin/Chettri/Newar			4	(5.1)			91	(9.9)
Place of residence								
Urban municipality			48	(60.8)			596	(64.7)
Rural municipality			31	(39.2)			325	(35.3)
Wealth quintile								
Lowest			7	(8.9)			46	(5.0)
Fourth			14	(17.7)			82	(8.9)
Middle			25	(31.6)			337	(36.6)
Second			20	(25.3)			237	(25.7)
Highest			13	(16.5)			219	(23.8)
Mother’s education								
None			15	(19.0)			106	(11.5)
Primary			21	(26.6)			187	(20.3)
Secondary			39	(49.4)			494	(53.6)
Tertiary			4	(5.1)			134	(14.5)
Father’s education								
None			14	(17.7)			103	(11.2)
Primary			19	(24.1)			133	(14.4)
Secondary			38	(48.1)			512	(55.6)
Tertiary			8	(10.1)			173	(18.8)
Mother’s occupation								
Housewife			75	(94.9)			866	(94.0)
Others			4	(5.1)			55	(6.0)
Father’s occupation								
Unskilled manual			53	(67.1)			528	(57.3)
Sales and services			18	(22.8)			276	(30.0)
Others			8	(10.1)			117	(12.7)
Father’s smoking status								
Yes			16	(20.5)			238	(25.8)
No			63	(79.7)			683	(74.2)
Sex of newborn								
Girl			37	(46.8)			459	(49.8)
Boy			42	(53.2)			462	(50.2)
**Maternal**								
Parity								
Nulliparous			51	(64.6)			484	(52.6)
Multiparous			28	(35.4)			437	(47.4)
Mode of birth								
Vaginal birth			72	(91.1)			762	(82.7)
Caesarean section			7	(8.9)			159	(17.3)
Pre-pregnancy BMI^b^								
Normal			59	(74.7)			690	(74.9)
Underweight			15	(19.0)			160	(17.4)
Overweight and obese			5	(6.3)			71	(7.7)
Number of ANC^c^								
≤ 3 visits			35	(44.3)			583	(63.3)
≥ 4 visits			44	(55.7)			338	(36.7)
SMM^d^								
Present			10	(12.7)			35	(3.8)
Absent			69	(87.3)			886	(96.2)
Obstetric haemorrhage								
Present			2	(2.5)			16	(1.7)
Absent			77	(97.5)			905	(98.3)
Hypertensive disorders in pregnancy								
Present			6	(7.6)			12	(1.3)
Absent			73	(92.4)			909	(98.7)
Severe management indicators								
Present			2	(2.5)			16	(1.7)
Absent			77	(97.5)			905	(98.3)

^a^ Expressed as median (interquartile range). Skewed to the right

^b^ *BMI* body mass index

^c^ *ANC* antenatal care

^d^ *SMM* severe maternal morbidity

The 20 independent variables associated with NNM were analyzed using simple logistic regression analysis (Table 3).

**Table 3:** Associated factors for neonatal NNM using simple logistic regression

Variables	Crude OR	(95% CI)	Wald Stat^a^ (df)^b^	P value
Mother’s age (year)	0.93	(0.87, 0.99)	4.23 (1)	0.040
Age of marriage (year)	0.94	(0.86, 1.04)	1.42 (1)	0.232
Duration of marriage (year)	0.95	(0.89, 1.02)	1.75 (1)	0.186
Ethnicity				
Muslim	2.53	(1.31, 4.88)	0.01 (1)	0.006
Terai/Madhesi	1			
Dalits	1.33	(0.70, 2.53)	0.78 (1)	0.376
Janajati	1.02	(0.53, 1.95)	0.00 (1)	0.951
Brahmin/Chettri/Newar	0.59	(0.20, 1.75)	0.88 (1)	0.376
Place of residence				
Urban municipality	1			
Rural municipality	0.84	(0.52, 1.35)	0.49 (1)	0.482
Wealth quintile				
Lowest	2.05	(0.84, 5.01)	2.48 (1)	0.115
Fourth	2.30	(1.14, 4.62)	5.49 (1)	0.019
Middle	1			
Second	1.14	(0.62, 2.10)	1.71 (1)	0.679
Highest	0.80	(0.40, 1.60)	0.40 (1)	0.527
Mother’s education				
None	1.79	(0.95, 3.37)	3.28 (1)	0.070
Primary	1.42	(0.81, 2.48)	1.54 (1)	0.215
Secondary	1			
Tertiary	0.38	(0.13, 1.08)	3.32 (1)	0.378
Father’s education				
None	1.83	(0.96, 3.50)	3.34 (1)	0.067
Primary	1.92	(1.07, 3.45)	4.85 (1)	0.028
Secondary	1			
Tertiary	0.62	(0.28, 1.36)	1.41 (1)	0.236
Mother’s occupation				
Housewife	1			
Others	0.84	(0.29, 2.38)	0.11 (1)	0.743
Father’s occupation				
Unskilled manual	1			
Sales and services	0.65	(0.37, 1.13)	2.32 (1)	0.127
Others	0.68	(0.31, 1.47)	0.95 (1)	0.328
Father’s smoking status				
No	1			
Yes	0.73	(0.41, 1.28)	1.19 (1)	0.275
Sex of newborn				
Boy	1			
Girl	0.89	(0.56, 1.40)	0.26 (1)	0.609
Mode of birth				
Vaginal birth	1			
Cesarean section	0.47	(0.21, 1.03)	3.55 (1)	0.060
Pre-pregnancy BMI				
Normal	1			
Underweight	1.09	(0.60, 1.98)	0.09 (1)	0.761
Overweight	0.82	(0.32, 2.12)	0.16 (1)	0.687
Parity				
Nulliparous	1			
Multiparous	0.61	(0.38, 0.98)	4.14	0.042
Number of ANC				
≤ 3 visits	1			
≥ 4 visits	0.73	(0.45, 1.16)	1.78 (1)	0.181
SMM				
Absent	1			
Present	3.67	(1.74, 7.72)	11.72 (1)	0.001
Obstetric haemorrhage				
Absent	1			
Present	1.47	(0.33, 6.51)	0.26 (1)	0.612
Hypertensive disorders of pregnancy				
Absent	1			
Present	6.23	(2.27, 17.07)	12.63 (1)	0.000
Severe management indicators				
Absent	1			
Present	1.47	(0.33, 6.51)	0.26 (1)	0.612

Note: *BMI* body mass index, *ANC* antenatal care, *SMM* severe maternal morbidity

^a^ Wald Statistics

^b^ Degree of freedom

Among 20 independent variables, ethnicity, wealth quintile, mother’s education, father’s education, father’s occupation, father’s smoking habit, mother’s age, age of marriage, duration of marriage, parity, mode of birth, number of ANC visits, hypertensive disorders of pregnancy and SMM were identified as associated variables with P < 0.3. These variables were included in multiple logistic regression analyses.

Mother’s education, parity, mode of birth and SMM were found to be significantly associated with NNM. Mothers without formal education (aOR 2.16; 95% CI 1.13–4.14) were at higher odds of experiencing NNM than those with secondary education. Multiparous mothers (aOR 0.52; 95% CI 0.39–0.86) were less likely to encounter NNM than nulliparous women. Newborns born by cesarean section were less likely to be NNM cases (aOR 0.44; 95% CI 0.19–0.99) than neonates born vaginally. Similarly, mothers with SMM were at higher odds of giving birth to an NNM infant (aOR 4.52; 95% CI 2.07–9.84) than those without (Table 4).

**Table 4:** Associated factors for NNM using multiple logistic regression analysis

Variables	aOR	95% CI	Wald stat ^a^ (df)^b^
Education of women			
None	2.16	(1.13, 4.14)	5.40 (1)
Primary	1.43	(0.81, 2.53)	1.57 (1)
Secondary	1		
Tertiary	0.38	(0.13, 1.10)	3.18 (1)
Parity			
Nulliparous	1		
Multiparous	0.52	(0.32, 0.86)	6.53 (1)
Mode of birth			
Vaginal birth	1		
Cesarean section	0.44	(0.19, 0.99)	3.89 (1)
SMM status			
Absent	1		
Present	4.52	(2.07, 9.84))	14.43 (1)

Note: *SMM* severe maternal morbidity

^a^ Wald Statistics

^b^ Degree of freedom

Note. No significant interaction; no multicollinearity problems model assumptions met; no influential outliers

## Discussion

The prevalence of NNM was 79 per 1,000 live births in Koshi Hospital, Nepal, using pragmatic and management criteria. Multiparity and cesarean section were associated with a decreased likelihood of NNM. SMM and mothers with no formal education were associated with an increased risk of NNM.

Consensus lacks a standardized period in which NNM is agreed to occur across countries, making it difficult to compare NNM-rates between studies. Some studies have used a near-miss period of 0–6 days [7, 10, 13, 22, 23], while others have utilized 0–27 days [15, 17, 19, 20, 24, 25]. Kale et al*.* recommend extending extrauterine life from seven to 28 days to increase the sensitivity of near miss criteria. However, a decrease in sensitivity was found when it was applied to 0–364 days [26]. In the current study, a period of seven days was used because four-fifths of neonatal deaths still occur within the first week of life, with one quarter taking place in the first 24 h [27].

In this study, the prevalence of NNM was within the range of previous studies of 45.1 to 72.5 per 1,000 live births that used the exact definition proposed by Pileggi-Castro, et al*.* [7, 13]. A population-based study in Nepal reported an NNM prevalence of 22 per 1,000 live births, which is much lower than our hospital-based study [18]. Possible reasons may be due to hospital versus population-based study, differences in NNM criteria used, and study settings [15, 20].

The prevalence of NNM was 87.6 per 1,000 live births in two studies from India, using only pragmatic criteria and a survival period of 28 days [19, 20]. It is higher than the 65 per 1,000 live births of NNM prevalence using pragmatic criteria only in our study. A survival period of 28 days versus seven days; increased sensitivity to enroll NNM cases due to the more extended survival period.

Multiparity was associated with a decreased likelihood of NNM, similar to other studies [17, 25]. However, studies from southern and northern Ethiopia reported multiparity as a risk factor for NNM [28, 29]. A recent prospective cohort study in Ethiopia reported grand multiparity as a risk factor for perinatal mortality among women with MNM [30].

Both nulliparous and grand-multiparous mothers were at high risk of developing complications during birth, which places neonates at risk of adverse outcomes [22, 31–33]. Nulliparity among mothers ≥ 35 years was a risk factor for adverse perinatal outcomes [34, 35]. Neonates born to advanced aged nulliparous women had a higher likelihood of admission to NICU [35–37]. However, in our study, the proportion of women aged ≥ 35 years and with > 4 children were small, lacking the power to draw further conclusions.

Prior studies have shown that firstborn infants are at higher risk of neonatal mortality than second- or third-borns [38, 39]. However, in some studies, parity was not shown to be associated with neonatal mortality [40].

Elsewhere, a higher risk of NNM among women undergoing cesarean section has been demonstrated [9, 25, 28, 41, 42]. In recent studies in India and Ethiopia, a direct association could not be established, although NNM occurred more frequently in women who underwent cesarean birth [7, 20]. Contrary to these studies, the cesarean section in our study was associated with a lower likelihood of NNM. In support of this finding, cesarean section reduced neonatal mortality in preterm births in the United States [43]. A study in the Gambia found that in women with MNM risk of stillbirth among vaginal birth increased four-fold compared to cesarean birth [44]. It is easy to understand because when stillbirth has occurred before any intervention is performed, there is generally no need for a cesarean section.

WHO recommends cesarean section only when medically necessary and recommends an upper population limit of 15% [45]. In our hospital-based setting, cesarean birth occurred in 17% in Koshi Hospital, which is higher than 12% in public hospitals in Nepal [4]. The proportion of cesarean sections performed in mothers with SMM was two times higher than in mothers without SMM (31% versus 16%). Previous literature showed SMM to be significantly associated with higher percentages of cesarean section and higher numbers of preterm birth than in women without SMM [46, 47]. An increase in fetal deaths and higher numbers of babies admitted to NICU for more than seven days was found together with increased numbers of cesarean birth [48]. A systematic review and meta-analysis showed that maternal and perinatal outcomes were often linked [49]. Mothers at high risk of maternal complications often gave birth by second-stage cesarean section to babies with low Apgar scores at 5 min. They were more likely to be admitted to NICU than after cesarean section during the first stage of labor [49].

Risks of intraoperative complications and hemorrhage following cesarean birth are high in low- and middle-income countries [49]. Timely intervention can prevent adverse neonatal outcomes among women with MNM [49, 50]. If selectively performed among mothers of fetus with a greater likelihood of being born alive, Cesarean section could be a confounder [51]. Overall, however, a lack of consensus exists in the literature that neonatal mortality and morbidity are higher in infants born by cesarean section than vaginal birth [49, 52–55].

Prior studies have not established a significant association between NNM and maternal education [7, 17, 20, 28, 41]. However, a universal association between maternal education and neonatal mortality, especially in low-income countries, has been demonstrated and supports our study findings [39, 53, 56, 57]. In addition, educated mothers more likely to have a higher socioeconomic status, have better knowledge of healthy behaviors, have a more informed approach to self-care, make better health-related choices and utilize healthcare appropriately [31, 58].

Our study found an association between SMM and NNM, consistent with a study in Ethiopia [7] but contradictory to one study in Brazil [59]. Very few studies, however, have explored the association between SMM and NNM. One study showed a strong association but with a lack of precision (OR 17.15; 95% CI 1.85–159.12), whereas others have not demonstrated a significant association between MNM and NNM [17, 42]. Mixed associations existed between obstetric hemorrhage and hypertensive disorders during pregnancy and NNM in southern Ethiopia [28] and Brazil [25]. In support of our study, an association between MNM and higher rates of adverse perinatal outcomes was found [44, 51, 60, 61]. Tura et al*.* claim that adverse perinatal outcomes among women with severe acute maternal morbidity (SAMM) are self-evident given the fact that SAMM is identified using severe clinical criteria along with organ dysfunction [30].

Among women with SAMM, also NNM is higher [22, 62–64]. A considerable number of newborns with low birth weight and neonatal hypoxia were born to women with MNM [51, 64]. A two-fold increase of stillbirths was found among women with more than two complications in the Gambia [44]. Similarly, maternal complications have been shown to play a role in the underlying causes of neonatal deaths [39, 65]. Therefore, early screening for poor maternal conditions during ANC and appropriate management of intrapartum complications is crucial to reducing NNM.

The current study did not establish any association between ANC and NNM, unlike a study in southern Ethiopia, where adequate ANC visits were associated with less NNM [28]. Attending ≥ 4 ANC visits was protective, whereas inadequate ANC visits increased neonatal mortality and adverse birth outcomes [63]. Possible explanations for non-association in our study were that only a quarter (24%) of women in Nepal received all seven components of ANC [66]. The majority of Nepal public institutions lack essential ultrasonography and laboratory facilities (blood and urine testing). Most pregnant women only receive health education, iron supplementation, blood pressure measurements, and anti-tetanus toxoid [66]. Secondly, there is poor compliance by pregnant mothers with ANC advice [67]. Hence, women with or without attending ≥ 4 ANC visits did not show any association with NNM.

With advancing maternal age, the prevalence of pre-existing conditions appears to increase, as does the risk of cesarean birth, contributing to increased fetal risks [68]. Advanced maternal age and younger age (< 20 years) were significantly associated with NNM [17, 29]. Secondary analysis of the WHO multi-country survey on maternal and newborn health showed that advanced maternal age significantly increased the risk of perinatal deaths [68]. No association, however, was established between maternal age and NNM in our study.

## Limitations

The findings of our study from a single referral hospital in Nepal may be generalized in similar study settings. Seventeen of the 44 neonates who required admission to NICU were self-referred to private hospitals with unavailable data. Multiple pregnancies were treated as single births. Firstborn neonates’ medical records were analyzed, which must have reduced the estimate of NNM prevalence. The date of the last menstrual period was used to calculate gestational age, possibly introducing incorrect estimations due to recall bias.

## Recommendations

The study of NNM deserves more attention as it has the potential to contribute towards reducing neonatal mortality. The NNM criteria should be used for up to 28 days of the neonatal period to increase its sensitivity. The higher number of cases could provide superior information regarding the pathway that leads to morbidity and death. In low-income countries, the unacceptably high neonatal mortality has to be assessed by a clinical audit of adverse outcomes. Future studies should standardize NNM criteria as different definitions were in use, limiting comparison across countries. Further studies can specifically explore the association of multiple pregnancies with NNM.

## Conclusion

The prevalence of NNM was 7.9%. Neonates of mothers with SMM were at increased risk of NNM; conversely, cesarean section, multiparity, and maternal secondary education were associated with reduced NNM. Healthcare providers should be aware of maternal factors associated with NNM. These obstetric factors, if screened earlier during pregnancy with appropriate interventions, will benefit newborn health. Strengthening facilities and healthcare providers’ skills, not only of NICU, can increase neonatal survival.

## Data Availability

The data is available upon request to the corresponding author.

## Abbreviations

SDG: Sustainable Development Goal
NNM: Neonatal near miss
WHO: World Health Organization
NICU: Neonatal intensive care unit
MNM: Maternal near miss;
aOR: Adjusted odds ratio
CI: Confidence interval
BMI: Body mass index
APGAR: Appearance, Pulse, Grimace, Activity, and Respiration
ANC: Antenatal care
SMM: Severe maternal morbidity
SAMM: Severe acute maternal morbidity
